# After the Pandemic: New Responsibilities

**DOI:** 10.1093/phe/phab008

**Published:** 2021-05-17

**Authors:** Neil Levy, Julian Savulescu

**Affiliations:** Uehiro Centre for Practical Ethics, University of Oxford

## Abstract

Seasonal influenza kills many hundreds of thousands of people every year. We argue that the current pandemic has lessons we should learn concerning how we should respond to it. Our response to the COVID-19 not only provides us with tools for confronting influenza; it also changes our sense of what is possible. The recognition of how dramatic policy responses to COVID-19 were and how widespread their general acceptance has been allowed us to imagine new and more sweeping responses to influenza. In fact, we not only can grasp how we can reduce its toll; this new knowledge entails new responsibilities to do so. We outline a range of potential interventions to alter social norms and to change structures to reduce influenza transmission, and consider ethical objections to our proposals.

## Introduction

Intellectually—as well as socially, economically and of course medically—we are all currently gripped by the COVID-19 pandemic. That is both appropriate and inevitable, given its stakes and the transformative effect it is having on the world. In this article, however, we urge the importance of stepping back and learning lessons about how we should respond to the world after the pandemic passes. We focus on seasonal influenza. We will argue that our response to COVID-19 has provided us with a new set of tools for responding to influenza, and as a result, we have acquired new responsibilities for applying these tools.

We will begin by briefly setting out the context in which we find ourselves, in the midst of the pandemic. We will then turn to the on-going challenge of seasonal flu, describing the extent of the problem it poses before putting forward proposals, many inspired by responses to COVID-19, for addressing it. The proposals are of two broad types: changes in structures, especially structures within the power of government and other regulators, and changes in norms. By changes in structures, we have in mind changes to regulatory frameworks, taxation law, policies governing education and employment: for example, we advocate paid parental leave to allow for the care of sick children, incentives for vaccines, moving to online education when a school suffers an outbreak and so on. By changes in norms, we have in mind changes to social attitudes to illness and those who are ill; our central example is our attitude toward those who continue to work when ill.

We will then examine the ethics of implementing these proposals. In common with most other writers on public policy, we will assess their justifiability by reference to their costs and benefits, using a very broad metric for what counts as a cost and a benefit. We will examine the similarities and differences between COVID-19 and influenza. The differences between them entail differences in the kinds of responses warranted. Unsurprisingly, some of these differences entail that more radical measures are justified in response to COVID-19 than to influenza. More surprisingly, in some cases, they have the opposite implication.

## Ethics in a Crisis

Applied ethicists typically work with empirical data they have not themselves generated: facts about how interventions work, about how societies change in response to new technologies and so on. When it comes to COVID-19, ethical reflection is hampered by the fact that the science is new and in flux. We are forced to make assumptions about the virus and its properties, and these assumptions may be wrong. We still do not have a good idea of the infection fatality rate of the virus: the number of people who will die after contracting it. Initial figures were 0.66 per cent (Verity *et al.*, 2020), but more recently, the Centre for Evidence-Based Medicine estimated that it could be as low as 0.1 per cent.^1^ Nor do we have any reliable estimate on the extent of morbidity in survivors. Without this information, it is difficult to produce a ballpark estimate of the benefits of many public health measures.

For instance, we are unable to estimate how many lives overall would be saved by economic shutdowns. The magnitude of the benefits is difficult or impossible to measure, given the current state of knowledge and the magnitude of the costs are also unknown. The lockdowns have resulted in a recession that seems certain to be long-lasting and severe, and recessions themselves have significant effects on morbidity and mortality. The 2008 recession seems to have resulted in at least 10,000 extra suicides in Europe and North America (Reeves *et al.*, 2014) and more than 250,000 extra cancer-related deaths in Organisation for Economic Cooperation and Development (OECD) countries (Maruthappu *et al.*, 2016). On the other hand, there is evidence that *during* a recession, all-cause mortality tends to fall (Ballester *et al.*, 2019). The apparent conflict between these findings remains unresolved. Perhaps recessions result in a spike in mortality, but that spike is delayed. Moreover, little is known about the effects of a recession in the developed world on the developing world (Peeples, 2019), though some forecasts have been dire (Ahmed, 2020). Engaging in responsible assessment of interventions in the face of these unknowns is, to say the least, challenging.

These problems may be inevitable for us, right now. In the context of a pandemic, we are forced to trade reliability for speed to some degree. However, in the face of these uncertainties, there is also a case for stepping back and thinking about the lessons the pandemic has taught us already and what their implications are for life after the crisis passes. We know a great deal about other common diseases, and we can reflect on what changes the pandemic might and should bring to our response to them.

In focusing on influenza, we do not wish to be seen to give comfort to those who assert that COVID-19 is just, or just like, the flu. While how much worse it is than influenza is currently unknown, that it is worse—that is, that it can be expected to be deadlier, whether due to a higher infection fatality rate or a higher infection rate (due to lack of prior immunity) or both—cannot be doubted. Even on the current lowest estimates of its infection fatality rate (0.1 per cent), it is roughly 10 times as bad as the flu. Rather than downplaying the seriousness of COVID-19, we want to emphasize the deadliness of influenza: its costs in mortality, morbidity and in social and economic terms. While these costs are surely lowered than COVID-19 in annualized terms, they are very significant.

Globally, influenza kills between 290,000 and 650,000 people every year (WHO, 2018). Since vaccinations, hospital care and good general health are all protective, many of these deaths occur in the developing world (>95 per cent of the children who die are in developing countries). But the burden of disease is significant in developed countries too. The Centers for Disease Control and Prevention estimates that the 2018–2019 flu season resulted in more than 34,000 deaths in the USA. This includes 136 children with confirmed diagnoses, but the CDC notes that influenza-related paediatric deaths are likely underreported (CDC, 2020b). The 2018–2019 flu season was rated as moderate. The previous flu season was much worse; it resulted in an estimated 61,000 deaths (CDC, 2019).

While COVID-19 will kill many more people than any seasonal flu (at time of writing, its global toll is over 1.5 million and cases are mounting at a rate of 600,000 per day) the *cumulative* toll from flu is very much higher. It is possible that in the future influenza’s toll will greatly outstrip COVID’s. That depends, in important part, on the future trajectory of COVID. It may be eliminated through a vaccine or through the development of herd immunity. Or it may become endemic, like other coronaviruses. If it becomes endemic, its virulence may fall, again like other coronaviruses. There are possible futures in which the virus remains a significant killer, and others in which it kills few or none. In most of these scenarios, influenza will be a bigger killer than COVID over time. Of course, in all these scenarios, it will also continue to be a major economic and social burden—it is estimated to cost an average of $11.2 billion annually in the USA alone (Putri *et al.*, 2018). Whether or not we manage to defeat COVID or learn to live with it, efforts to address the flu will remain important.

We will suggest that the world’s experience of the pandemic has altered our responsibilities when it comes to influenza. The world after the pandemic will not be the same as it was beforehand. Some of the changes may make responding to influenza easier. Just as importantly, our response to the pandemic has transformed our sense of what is possible. It has shown us how dramatic transformations of daily life to fight disease can be, and what we can achieve. Who would have thought a year ago that mask-wearing would be the norm?

These transformations, in our sense of what is possible and in our knowledge of how infectious diseases can be fought, confer new responsibilities on us because the scope of responsibility is sensitive to what potential bearers of responsibility know or truly believe (Robichaud and Wieland, 2017). If such bearers are aware that future harms can be confidently predicted and that there are actions available to them that can significantly reduce these harms (without entailing larger costs), then they have a prima facie responsibility to take these actions. This is a responsibility they can avoid only if others are better placed than they are to take these actions, or if those on whom the harms will fall deserve them. Neither of these escape clauses are available with regard to influenza. Governments, workplaces and individuals are now in a position to grasp the harms that stem from influenza and to take effective action to reduce them significantly. Consequently, the pandemic has transformed our responsibilities, including our responsibility to fight influenza.^2^

Combatting seasonal flu (and other infectious disease) more effectively than in the past will require a change in social norms, as well as in economic and social structures. Below we outline some of the changes that might be warranted by the seriousness of the burden it represents. We caution that in the space available, we are unable to offer full defences of any specific proposal. We do not doubt that some will face significant objections we have not contemplated and that other proposals may prove to be better justified. We offer them as plausible suggestions and to spur further work on the topic.

## Changes in Norms

Presenteeism—continuing to work when ill or injured—is very common. In 2010, 40 per cent of employees across 34 European countries reported working at least one day in the past 12 months while sick (Kinman, 2019); other studies report much higher rates (Lohaus and Habermann, 2019). This may occur for multiple reasons. A central impetus stems from a rational assessment of the potential costs of taking time off. Self-employed people may simply go unpaid; those on zero-hour contracts risk losing their shifts permanently and those with apparently more stable employment risk being seen as unreliable (Kinman, 2019). Workers with more senior or secure roles may feel an obligation to colleagues to do their fair share of the work (Dew *et al.*, 2005), or a need to set an example for less senior staff (Kinman, 2019). Workers’ self-conceptions as team players may also encourage presenteeism (Kinman *et al.*, 2019).

As the pandemic has now brought home to all of us, the workplace is an ideal environment for the spread of infectious disease, because it brings many people in close proximity indoors and for an extended period of time, often with poor ventilation. This fact ought to encourage employers to change the norms and (dis)incentives that contribute to presenteeism. Sick employees are less productive than healthy ones, so encouraging them to stay at home might not represent a large loss in workplace productivity. Moreover, those who are suffering from an infectious disease are at risk of spreading it, thereby lowering the productivity of those around them. They are also more likely to make mistakes or cause accidents, sometimes at an extremely high cost. For all these reasons, the costs of presenteeism to the workplace may be higher than the benefits of having sick employees come to work; perhaps much higher (Garrow, 2016). This fact gives employers and senior managers a reason to discourage it.

Of course, costs to individual workplaces are not the only costs that weigh in favour of discouraging presenteeism. There are also costs to the wider society: health care costs, lost productivity in other workplaces as the disease spreads to chance contacts on the way to and from work and to the family members of fellow workers, and so on. These additional costs provide incentives to governments and other regulators to encourage or mandate better protections for workers. These might take the form of guaranteed sick days and a requirement that they are taken, and protection against losing shifts or employment due to taking them. Government might go further, itself paying for sick days, directly or by payment or tax reduction to the employer. A number of governments around the world currently offer compensation for people who must self-isolate due to COVID-19 and who will thereby lose income. Such a scheme might be extended to other infectious diseases.

There is a risk of a kind of ‘moral hazard’ or perverse incentive with policies like these: people will have an incentive to call in sick, with employers having less incentive to police sick leave. Clearly, there would need to be verification procedures, which themselves might involve negative externalities (e.g., visits to physicians). Getting such a policy right will be difficult but not insurmountable.

COVID has taught us, moreover, that the costs to government and to workplaces of modified work practices can be lower than we might have thought. Technological changes ensure that in many occupations, absent workers can continue to contribute.^3^ While we do not advocate entirely replacing leaves of absence with distance work, because people may require complete breaks for mental and physical health, it is often the case that working from home is a good option for employee as well as employer. It enables the employee to continue to feel productive and connected to the workplace and costs to their health may be zero or very close to it. Working from home also has positive externalities, such as a reduction in pressure on roads and emissions from car use. Extended use might have significant costs, such as a sense of isolation, but its occasional use for a short period does not pose these problems.

Pre-pandemic, unwell people would often be congratulated by bosses and co-workers for their fortitude in continuing to come into the workplace. This is a theme of many advertisements for cold and flu medications: they are alleged, for example, to allow the person to ‘soldier on’.^4^ We argue that these norms should be reversed. Workplaces should discourage employees from working when unwell, inverting the current incentive structures. Senior management can model this behaviour; doing so seems to be effective in changing workplace culture (Dietz *et al.*, 2020).

Norm change is not the responsibility of workplaces alone. We have already mentioned ways in which government can support workplaces and employees. Government has a broader role to play in changing norms. There is evidence that advertising campaigns, backed up by incentives and disincentives, can change behaviours and attitudes (Snitow and Brennan, 2011). These messages may be seen as *nudging* norm change. Nudges are canonically understood as ways in which agents’ choice architecture—the context in which choices are made—is altered by changing the environment (Thaler and Sunstein, 2008). But if we understand them more broadly, as ways of influencing choice that offer neither explicit incentives nor explicit reasons for choice, then they may include ways in which options are presented. A number of countries now require tobacco companies to sell cigarettes in packs with prominent and specific health warnings and confronting images of damaged organs and other health problems displayed. They thereby combine explicit messaging with what might be seen as a nudge. Governments might leverage such nudges to change social norms around presenteeism.

The pandemic has brought about a widespread appreciation for how even those at low risk of serious health complications have an important role to play in protecting vulnerable individuals and health systems. Nudging much more widespread use of vaccines might change social norms by building on this appreciation in the context of influenza. At the time of writing, there are several very promising candidates for a vaccine against COVID-19, and the UK has given emergency approval to two of them. However, vaccine development is far more advanced for the flu than for COVID. Because the influenza virus (or more properly viruses) mutates from year to year, the vaccine must be continually updated and effectiveness is, on average, moderate (Castilla *et al.*, 2016). Moreover, the protective benefits of vaccines are smaller for some of the most vulnerable people (Henry *et al.*, 2019). Nevertheless, the vaccine already reduces the burden of the disease very significantly and wider usage would certainly have a large effect, but uptake remains low (Wang *et al.*, 2018), even among health professionals (Wilson *et al.*, 2019). Myths about vaccines are a significant barrier to uptake (Giubilini, 2019), as is a lack of personal vulnerability. Conversely, a sense of responsibility to others is a significant predictor of uptake (Wilson *et al.*, 2019).

Finally, other nations might encourage and adopt norms that are more common in East Asia. Mask wearing is common, especially when symptomatic in some East Asian countries. Moreover, in at least some countries, it is associated with frequent hand washing (Wada *et al.*, 2012). It remains unclear whether the benefits of mask wearing are large enough to warrant a social norm of wearing them, but hand washing is certainly effective and should be promoted. Social distancing should also be practiced when symptomatic.

## Changes in Structures

As we have already emphasized, changes in norms will be much more effective if messaging, modelling and nudging is backed up by changes in regulatory structure. We cannot expect appeals to altruism to be effective among those at risk of losing their jobs. Not only may structural alterations facilitate compliance with the norms; they also amplify the message by communicating a sense that the issue is being taken seriously.

A part of Japan’s success in the COVID pandemic has been attributed to its established system of surveillance of citizens during flu epidemics, together with rapid contact tracing (Du and Huang, 2020). Development of rapid diagnostic tests and contact tracing could also be employed during flu pandemics to pre-empt spread through schools and workplaces. A contact tracing app might also play a role. Here, we need to learn from the failure of the UK system and of similar systems around the world (Cellan-Jones and Kelion, 2020). One issue they face is that their use is voluntary. Mandatory contact tracing apps would be effective at promoting public health, though they face community concerns relating to privacy (Cho *et al.*, 2020). There may be ways to design apps to minimize such problems (Yasaka *et al.*, 2020).

In addition to the structural changes mentioned above, there are a number of other ways in which regulators might discourage presenteeism, encourage self-isolation and vaccine uptake. Temporary school closures have been effective in reducing flu transmission (Jackson *et al.*, 2016), although the optimal timing and duration of such closures remains unclear (Jackson *et al.*, 2013). School closures can be mandated by the government when outbreaks exceed a certain proportion of students. Alternatively, individual pupils might be required to stay home when symptomatic and therefore likely to be most infectious, or when they have been in contact with infected individuals. Such mandates will be more effective if combined with other measures that support parents when their routines are disrupted by caring responsibilities. Again, government has an important role to play in this regard. The sick leave entitlements needed to reduce presenteeism might be accompanied by parental or carer leave.

Since a large proportion of the time children are self-isolated or schools are closed, they will be well enough to continue schooling, governments should also take steps to reduce the costs to children in terms of lost educational opportunities. Again, we can easily build on measures introduced during the pandemic, especially online learning. This may be delivered through a mixture of appropriate software (government might subsidize the development of such software) and teacher instruction. Note that such instruction might be more burdensome for teachers than online teaching during the pandemic when most pupils are at home, since it would necessitate separate instruction for self-isolating pupils and an in-person class. Teachers cannot be asked to do double duty without compensation; alternatively, dedicated and additional staff might deliver online instruction.

Changes in structures can also help to increase vaccine uptake. Obviously, vaccines can be provided free of charge. Many countries provide flu vaccines free to the vulnerable, but expanding provision to everyone who can benefit from the vaccine might be advisable: the elderly are less well protected by the vaccine, and the indirect protection conferred by less virus circulating might be more effective at protecting them. Vaccination of children may be the best way to protect the elderly and other vulnerable groups (Bambery *et al.*, 2018). A major reason why parents do not vaccinate their children is simply inconvenience: a lack of time, inaccessibility of clinics and so on (Betsch *et al.*, 2015). Obviously, these barriers can be overcome by structural changes. Vaccines could be delivered at central locations, offered to children at their schools and so on.

Many states allow parents to opt-out of otherwise mandatory vaccinations on religious or ‘philosophical’ grounds (Diekema, 2014). The grounds for these exemptions are often dubious and they are used as cover for belief in conspiracy theories. Attention should be paid to narrowing or eliminating these exemptions. Consideration should be given to making vaccines mandatory, on the grounds that unvaccinated people represent a risk to others. Disincentives could include withholding child benefits (No Jab, No Pay as occurs in Australia) or fines (as in Italy). We should, however, adopt the least or less restrictive option and incentives may generate the benefits of vaccination without the need for coercion (Savulescu, 2020). Other ways of increasing vaccination rates can also be ethically justified. For example, competent adolescents can consent to their own vaccination. Opt-out policies of school vaccination enjoy wide community acceptance, and can be expected to increase uptake significantly (Giubilini *et al.*, 2019).

There are many other things governments and regulators can do to increase vaccine uptake, to encourage self-isolating and reduce spread of infectious diseases. If masks are judged to be sufficiently effective, they might be provided free of charge. Hand-washing stations and hand sanitizer can be provided. Initiatives to encourage working from home after the pandemic (e.g., appropriate tax incentives) could be pursued. More ambitious measures, and more controversial, might focus on the conditions that lead to novel pathogens, including (but not only) new strains of influenza. Zoonotic influenza like the avian and swine flu emerges from intensive farming. These farms might be regulated more tightly, or higher standards of hygiene mandated. Many philosophers believe that these farms are unethical on animal welfare grounds; these concerns might be an additional reason to prohibit such farms altogether (Pluhar, 2010).

**Table 1.** Differences between COVID-19 and seasonal influenza relevant to the assessment of the costs and benefits of measures designed to tackle each

Dimensions	COVID-19	Influenza	Implications
Severity	Moderate IFR 0.36–0.6 Long COVID in a minority of cases Strain on health system	IFR approximately 0.1 Almost all affected recover without long-term harm Usually little strain on health system	More costly interventions warranted for COVID than influenza
Effects on cognition	Significant in a large minority of cases, sometimes long-lasting	Moderate in the acute phase in almost all cases, very rarely long-lasting	More costly interventions warranted for COVID than influenza
Recurrence	Unknown	Seasonal illness, entailing the need to lock in measures that persist or can be reactivated	Longer lasting, more structural changes may be more warranted for flu than COVID
Riskiness of counter- measures	Costs and benefits of responses, including medical interventions, remain unknown	Better understanding of costs and benefits of responses; possibility of learning from the COVID experience about novel measures	May warrant locking larger and more permanent interventions for flu than for COVID
Familiarity	COVID is highly salient and novel	Flu is very familiar and recessive in thinking	May tend to cause an underestimation of the risks of flu relative to COVID (and absolutely)

## COVID-19 and Influenza: Similarities and Differences

We can *directly* transfer the lessons we have learned from the COVID-19 pandemic to influenza outbreaks only to the extent to which they resemble one another. It is equally obvious that there are differences between COVID-19 and influenza. While differences between the two viruses and how they manifest entail difficulties in directly transferring lessons from the first to the second, some lessons can be learned. Differences might be instructive, and responses to one kind of problem often provide useful guidance for responses to another. Reviewing these similarities and differences is essential for an assessment of the ethics of the responses we advocate (Table 1).

In common with most other writers on public policy, we take it that large-scale responses to a public health problem must have a favourable cost/benefit profile. In adopting this framework, we do not beg the question against non-consequentialist moral theories: we do not assume that either the costs or the benefits must be assessable in narrowly consequentialist terms. ‘Costs’ may refer to financial costs and the opportunity costs of expenditures; equally it may refer to losses of liberty or opportunities for self-cultivation. The same considerations apply to ‘benefits’. Allowing for broad and ecumenical measures of costs and benefits ensures the need for judgment in weighing costs and benefits. What weight does one assign to the restriction in liberty represented by, say, mandatory mask-wearing? How does one weigh that restriction against the risk of the loss of a life; indeed, how does one go about measuring the cost of a lost life? Should all lives count the same, or should lost lives be weighted for age or quality of remaining years? These judgements are *value* judgements. We take it that the need for such judgements is inescapable, and that there can be reasonable disagreements about them. However, we also believe that some disagreements would be *un*reasonable: a clear-eyed appreciation of the facts about the diseases and possible responses provides us with guidance that sets the bounds for what kinds of responses are appropriate.

### Severity

COVID-19 can be reasonably regarded as a public health emergency. ‘Emergency,’ here, is intended to indicate a problem of sufficient severity to warrant highly costly responses. Emergencies come in degrees: COVID-19 is not reasonably regarded as what we might call a *supreme* emergency, which warrants actions that would be unthinkable at other times. We do not have a precise cut-off for when a disease might constitute a supreme emergency; clearly, however, COVID-19 is not a challenge on the scale of the Spanish flu or the bubonic plague, which would warrant more disruptive interventions.

At least in most years, influenza is reasonably regarded as a public health challenge that falls short of being an emergency. As the figures, we quoted above clearly show, it is certainly a serious challenge: thousands of people die from the flu every year. But measures designed to prevent flu deaths must be sensitive to the costs. Lockdowns are unlikely to be justified in most flu seasons, for two reasons. One involves a contestable value judgment: lockdowns may be unjustified to combat flu because the restriction of liberties involved is a cost that is high enough to outweigh the benefits. The second is more straightforwardly empirical: lockdowns have costs *measured in terms of health* (delayed visits to physicians, lack of exercise, mental health burden, increases in domestic violence), and the ill-health that results from a lockdown can be justified only if it saves more lives than it costs.^5^ Because the flu has a high cost, in terms of ill-health and death, somewhat restrictive measures may be justifiable, but less restrictive than those justified by COVID-19.

It is important to note, however, that mortality is not the only measure of severity. Both diseases are, obviously, unpleasant in their acute form, and the entailed suffering itself justifies measures to prevent and treat them. But COVID-19 is sometimes much more severe, even when it does not lead to long-term problems. More significantly, the phenomenon of ‘long COVID’—experiencing a range of health problems long after the virus has cleared the body—is a very significant cost. Post-viral fatigue is also reported with influenza, but the effects seem to be longer-lasting and more severe with COVID. Moreover, there is evidence of damage to the heart, kidneys and other organs (CDC, 2020b). These costs themselves justify more restrictive or costlier responses to the disease than might be justifiable on the grounds of infection fatality rate alone.

COVID also has much more dramatic effects on the capacity of the health system. In most years, the burden on hospitals from seasonal influenza is low to moderate, and few additional resources are needed. COVID may strain available resources and has significant costs beyond mortality and morbidity of sufferers, in the form of diverting of resources away from other patients and delayed diagnoses. Surge capacity may be needed for COVID that would never be called on for flu.

### Riskiness of countermeasures

The more severe a problem, the greater the urgency to address it and the higher the costs that can be justified. However, different responses carry probabilities of success and potential for harm. Decision-making in the face of COVID-19 presents problems of risk in the technical sense: that is, the probability of success and the likelihood of unintended consequences are unknown. We can be reasonably confident, for example, that lockdowns have negative effects on mental health and on economic activity, but the magnitude of these effects remains unknown.

We should favour less costly responses over more, other things being equal, but in situations of risk, we cannot assess the costs with any degree of precision. The severity of COVID-19 entails that risks may be worth taking, but the lesser severity of influenza does not justify the same risks. However, the degree to which riskiness limits the type of measure we can employ against influenza may be much smaller than these considerations suggest. First, because influenza has been around much longer than COVID-19, vaccines are less likely to have side-effects that escape detection during trials (for instance, because they arise from unexpected interactions with medications or rare phenotypes). Secondly, to the extent we aim to transfer responses from COVID-19 to influenza, we will be able to benefit from our experience with the former. In effect, the current pandemic will serve as a trial of measures we may be able to utilize in the future. Paradoxically, this fact may entail that some measures are easier to justify as a response to influenza than to the more severe COVID-19 (conversely some measures may be shown to be ineffective or to have unacceptable costs).

### Likelihood of recurrence

Influenza is a seasonal illness. It is this fact that entails that it is important to implement measures that effectively respond to it: the annual toll from influenza is relatively small in most years, but the cumulative toll is very significant. An effective response to influenza puts in place measures that can be reactivated annually. It remains unknown whether COVID-19 will persist indefinitely as a seasonal illness. We have succeeded in eradicating only smallpox, but a number of other diseases are very well-controlled (e.g., polio). Changes to social structures and other measures that are systematic might therefore be harder to justify in response to COVID than to influenza. We are more confident that such measures will be required with regard to the latter than the former.

## Effects on Cognition

Fatigue and headache are common symptoms of influenza; these symptoms have cognitive effects. An acutely ill influenza sufferer cannot easily focus on a demanding task, for instance. This fact has direct implications for their productivity as workers, as well as their capacity to drive and, more generally, to make good decisions. COVID-19 usually causes symptoms that are similar. However, in an as yet unknown proportion of cases, COVID causes much more severe cognitive impairment. Moreover, ‘brain fog’—memory and concentration problems—may persist for months after clearing the virus (Amenta *et al*., 2020).

The depths and persistence of the cognitive impairments associated with COVID entail that discouraging work attendance and encouraging home isolation may be justified based on the prevention of harm to others. A brain fogged worker or driver may be a risk to themselves and to those around them. The generally milder impairments associated with influenza are more compatible with a quicker return to normal activities, especially once the infectious period has passed.

## Familiarity

People are more cautious with regard to rare and novel threats than to those that are familiar. It is, in part, for that reason that we may fear death from a terrorist attack or an accident at a nuclear power plant more than the much more common threats like Salmonella or respiratory illnesses arising from the inhalation of emissions from coal (Burns *et al.*, 2010). While some rare events may prove so costly we ought to insure against them despite their low likelihood, we often over-insure for rare but high-impact events, and underinsure for more likely but less dramatic events. We also underinsure for risks—like a pandemic—that are not salient to us. It is likely that these same factors influence our response to COVID-19 versus the flu.

COVID-19 is highly salient to us. It is also a novel threat. It is likely that it, and the threat of the next pandemic, now loom larger in our minds than is warranted, relative to other problems. It is very likely, to take a central example, that the current emergency has led us to pay too little attention to the climate crisis, which is more familiar but which is very likely to be more costly in the medium term. Flu, of course, is very familiar and not very salient for most of us, most of the time. We may therefore underinvest in responding to it (Table 2).

**Table 2.** Lessons from COVID-19 for future flu epidemics

Workplace	Disincentivize presenteeism by encouraging taking of paid leave or working from home when illness is suspected
Educational institutions	Targeted and limited school or class closures, with substitute online teaching
Public Health	Increasing influenza vaccination rate by mandating it, making it opt-out or the introduction of disincentives (such as withholding child benefits or fines) or incentives, such as payment
	Disease surveillance, development of rapid testing, contact tracing, with consideration given to making apps mandatory
Regulatory/legislative	Better protections for those who need to self-isolate; mandatory provision of paid leave
Individual	Development of a culture of self-isolation based on symptoms, a positive test or exposure to an infected individual
	Voluntary social distancing and possible mask wearing while symptomatic or in contact with an infected individual
	Regular hand hygiene
National/international	Greater surveillance and monitoring of farming and food production, and disease emergence globally

## Ethical Concerns

Since we advocate assessing public policy proposals by reference to their costs and benefits, the above considerations set the scene for a fuller assessment of the ethics of changes to norms and structures to combat influenza. In this light, measures aimed at combatting influenza are highly likely to be justified: the harms of influenza are large. We recognize, of course, these measures have economic costs. The provision of vaccines is costly, and measures to ensure adequate coverage for those encouraged to take time off from work, ranging from substitute teachers to childcare to the provision of infrastructure for online work and education would be more costly still. We would not attempt to quantify these costs here. However, these economic costs must be offset against their benefits. The economics of vaccines is complex (Postma, 2008), but there are grounds for thinking that the economic costs will be negative.

It is likely that the most controversial proposals will be those involving coercion. Coercion always requires justification, but we suggest that it may be permissible. Coercion is acceptable in public policy, we suggest, only when it satisfies the Millian harm principle: when it is the least restrictive or otherwise costly option available to prevent harm to *others*, rather than to the person coerced. Coercion is most justified when the harms averted are large and the costs to the person coerced small (Giubilini *et al.*, 2018); it is difficult to justify when the benefits accrue only to the person coerced or the harms to that person are larger than the benefits to others, or large enough to make their rescue ‘difficult’ (Giubilini *et al.*, 2018). The large direct and indirect costs of influenza seem to warrant some degree of coercion, although it is open to question how much can be justified.

Another broad-brush objection might focus on the legitimacy of what is sometimes called *social engineering*. The social engineering objection is an old one, dating back at least as far as conservative objections to Enlightenment proposals for social reform, and focuses on the unintended negative effects of centrally planned attempts to change society for the better (Støvring, 2014). While it is tempting to reject this kind of objection as mere status quo bias (Bostrom and Ord, 2006), to the extent to which it cautions us that all attempts at amelioration have unintended consequences, and it is likely that some of these consequences will be negative, it serves a salutary role. Similar to the ‘Playing God’ objection to radical biotechnology, it reminds us to proceed cautiously and seek the best inputs in designing interventions, and to be on the lookout for negative effects. We have attempted to be cautious in advocating specific proposals here, in light of the warning it provides. But we do not think any *general* objection from social engineering has any chance of success. Centrally planned projects for the amelioration of social ills have sometimes gone disastrously wrong: the shortages and waste that characterized the Soviet Union were probably at least partly due to rigid top-down control over the economy. But the history of public health features many successful examples of centrally planned and implemented interventions which together have done more to extend lifespans than have new developments in treatment. A famous example is the construction of the London sewers, in response to the threat of cholera. The works were mandated by an act of parliament, as were many later improvements in the treatment of sewage (Brewer and Pringle, 2015). The inflexibilities of Soviet central planning need not and should not be replicated by planners.

In light of the risk of unanticipated consequences that are sufficiently negative to warrant a rethink, it would be unwise to lock in structural changes until they have been sufficiently tested. Above we noted that the COVID pandemic provides us with an opportunity to learn about such consequences for many of the interventions we advocate. Those might be implemented with more confidence; others should be introduced more carefully and tentatively.

A more limited social engineering objection might object to attempts to change social norms. That may seem objectionable insofar as it might be seen as trying to change our thoughts, rather than (merely) our behaviours. Because thoughts are more intimate, such attempts may be seen as needing to satisfy a weightier burden of justification in order to be legitimate.

In its general form, we think this objection fails. It arises, we think, from awareness of a fact that some find disturbing: that how agents think is intimately shaped by their social circumstances, including the behaviour of those around them and their social environment. Social norms are already and inevitably shaped by these things, most powerfully during our childhood but to some degree across our entire lifespan. Our social norms are and will be shaped by the *kinds* of things we propose, like it or not, whether or not they are shaped by proposals like ours. Any objection to such shaping is nothing less than an objection to being social animals of the kind we are.

However, an objector might accept this point and nevertheless argue there is a crucial ethical difference between *designed* interventions into the kinds of prompts that shape norms and those that occur without design. This kind of objection is familiar from the literature on nudges: in reply to a common defence of nudges, that since they will occur anyway there can be no grounds for objecting to using them for the benefit of the nudged, several writers have urged we should be more wary of intentional than unintentional nudges (Kumar, 2016; Vallier, 2016). Kumar (2016) suggests that only intentional nudges can be manipulative. He suggests that they therefore threaten agents’ autonomy in a way that unintended interventions do not. A second objection might focus on the relationship that intentional interventions establish between interveners and those intervened upon. There is surely something objectionable in a small group of agents deciding what the norms of a much larger group should be.

We think that these objections point to genuine concerns, but there is no reason to think that they give us grounds to reject interventions aimed at changing social norms in general. Rather, they point to features of potential interventions that would give us a reason not to employ those particular interventions. It may be true that some interventions of the kind envisaged might limit autonomy. But whether they do will depend entirely on what impact they have on agents. Some such interventions leave agents’ capacity for reasoning intact and do not restrict agents’ options unduly. Unless one believes, implausibly, that it is a limitation on their autonomy that people face social disapproval for behaving antisocially, it is hard to see how the mere fact that interventions change incentives or norms limits autonomy.

We can compare the interventions we propose to interventions to increase altruism in other areas, for example, organ donation. We do not think encouraging organ donation is immoral. Indeed, there are good arguments to make it opt-out, or to give priority to organ donors, or remove the family veto over organ donation (Savulescu and Isdale, 2015). The UK has recently moved to an opt-out scheme for organ donation.

It *would* be objectionable for a small group of self-appointed agents to attempt covertly to engineer the norms of others and subvert democratic process. But that is not what we envisage. Interventions, whether aimed at norm change or structural change, should be carried out openly, with full explanation and justification of their rationale and purpose. We hope, indeed, that these explanations will themselves play a role in norm change: when people see that the reasons for changing norms are good ones, they will be readier to embrace them. While it is true that the structure of these interventions involves some people exercising power over others, this structure is inherent in governance. Those in power should explain how and why they implement their policies and be open to democratic control over their implementation.^6^

An example is the contrast between mandatory and voluntary educational/persuasive approaches to vaccination. Any scheme for vaccination—voluntary, incentivized or mandatory—should be accompanied by maximum public education to enable people to understand the basis of policy.

We mentioned above that worries about autonomy often focus specifically on nudges; since we advocate nudging as part of our proposals, it is important to address these concerns. The worry, in brief, is that nudges threaten autonomy because they bypass rational deliberation. Rather than give us reasons, they take advantage of the fact that we are ‘somewhat mindless, passive decision makers’, as Thaler and Sunstein (2008: 37) themselves put it. They rely, allegedly, on our intuitive but unintelligent biases, not rational thought. Since autonomy consists in rational self-government, nudges threaten autonomy (Bovens, 2008; Saghai, 2013; Wilkinson, 2013). We do not have space fully to respond to this concern here; instead, we point to a fuller discussion elsewhere (Levy, 2019). In short, we believe the objection goes wrong at the outset: nudges rarely bypass rational cognition. Rather, they (or at any rate typical nudges, including all the nudges typically cited in the literature) work by giving agents genuine, though implicit, evidence in favour of acting as they are nudged. Paradigmatically, they function as implicit recommendations. Nudges typically work by making some option salient to us; for example, selecting a default on an insurance policy makes it salient to us. In doing so, they recommend it to us, and we respond rationally in being guided by that recommendation. As many epistemologists have emphasized, testimony is a good source of evidence for agents and they ought to modulate their beliefs in its light (Coady, 1992; Lackey and Sosa, 2006).

We do not take this survey to be exhaustive of the ethical objections to the kinds of interventions we propose. But we are sceptical that objections are forthcoming that should lead us to abandon the project entirely. The reason for our scepticism is that any such objections face a high justificatory hurdle. They must show that the ethical costs of these proposals (or others, should they prove ineffective or unduly costly) are sufficiently high to justify abandoning proposals that are literally life-saving. The direct and indirect costs of influenza are so high, year on year, that any effective means of reducing them significantly has a great deal in its favour. Of course, there may be alternative means of securing the benefits our proposal aims at which are less ethically costly. We would welcome such proposals.

## Conclusions

For good reason, the attention of ethicists and other researchers is focused, right now, on the challenge of COVID-19. It is worth stepping back from the pandemic and its immediate impact, however, to reflect on what we have learned from our response to it and how what we have learned can guide us in responding to the challenges we will confront when (as we hope and expect) the crisis it represents recedes. We have suggested that our response to COVID-19 provides guidance as to what is possible and advisable in response to other infectious diseases and in particular seasonal influenza. Influenza is not the killer that COVID is—not usually, in any case—but its annual toll is large, and its cumulative burden exceeds COVID-19. Addressing it is not as urgent as addressing COVID-19 (the cumulative toll is not paid at once, of course, so if we delay a year or two, we will still be able to reap most of the benefits) but it nevertheless extremely important.

We have suggested that our response to the pandemic transforms our sense of what options we have. It shows us that truly dramatic interventions can be accepted by most people, if the need for them is explained, and therefore assures us that the less dramatic, but nevertheless significant, proposals we envisage are realistic. It also changes our sense of what our levers are. Moreover, we have argued that the new knowledge we have, of what kinds of interventions are possible and what their effects are likely to be, transforms our responsibilities. Once we know that we have the power to prevent significant harms, we acquire the responsibility to do so. This is a responsibility we can avoid only if others are better placed than we are to bear it, or if the harms will befall those who deserve them. While there are many individuals who escape responsibility in the first kind of way—those with few resources, for example, cannot reasonably be expected to alter their lives to help prevent flu—there are many others who cannot. Decision-makers in government and business cannot shirk their new responsibility to help prevent influenza, and most ordinary people have or will acquire a responsibility to play their part, once they are asked to and they are given the opportunity.

It is noteworthy, finally, that the kinds of proposals we envisage can be expected not only to play a role in reducing the annual toll of seasonal flu, they will also help to make the next pandemic less likely or less severe. The next pandemic might well be a flu virus; whatever it is, it is likely that having in place arrangements for self-isolation and working from home, for closing schools and paid sick leave, as well as norms that frown on working while ill and that encourage good hygiene, will slow its spread and reduce its toll. To that extent, we believe the proposals we urge should be high on our agenda.

It is worth acknowledging that there could be public resistance to our proposals. Note, however, that 50 per cent of Americans were against the introduction of mandatory seat belt laws. Now well over 70 per cent endorse them (Beck *et al.*, 2019). Seat belts are mandatory because they impose very little cost on the individual (the risk of death or serious injury from them is small) and the benefit to the individual (50 per cent reduction in mortality) and to society is great (in terms of reduction in the use of limited health care resources).

Public resistance is a good reason to abandon a policy, both because a lack of widespread support tends to render a policy ineffective and because in democracies the public has the right to settle policy questions. However, politicians should sometimes lead public debate rather than follow it.^7^ We urge that if the evidence supports the proposals we have outlined here, or alternatives that serve the same end, they should be implemented. We were confident that public sentiment will swing behind them.

## Conflict of interest

The authors declare they have no conflicts of interest.

## Footnotes

1 See https://www.cebm.net/COVID-19/global-COVID-19-case-fatality-rates. The US Canters for Disease Control and Prevention estimates an infection fatality rate of 0.26 per cent (CDC, 2020a).

2 Of course, it might have changed our responsibilities with regard to many other things too. We might have new responsibilities to prevent the emergence of other infectious diseases, for example (perhaps by regulating wet markets), or to change working conditions to combat the inequalities that have become stark during the pandemic. We do not claim that combating influenza is the only or even the most urgent new responsibility to emerge from the crisis.

3 Technological changes—the internet—also lower the costs of self-isolation in other ways, relative to previous decades. The food, goods and entertainment delivery services the internet enables, as well as the new means for staying in touch with friends and families, ensure that measures that would have been unthinkable just a few years ago are now practicable.

4 A common brand of Australian cold medication uses this as its slogan. Interestingly, in 2008 a complaint was brought against this company on the grounds that its advertising encouraged consumers to continue to work and socialize while unwell. The Australian Advertising Standards Board dismissed the complaint, finding that the advertisement did not contravene ‘prevailing community standards’. We suggest that that finding was correct, but illustrates the need for a change in the prevailing standards (see https://adstandards.com.au/sites/default/files/reports/388-08.pdf for the judgment).

5 While we stand by the claim that this kind of judgment is more straightforwardly empirical, it’s important to note that scientific values always themselves reflect values. Assessing the health costs of an intervention, for example, requires a careful calibration of evidential thresholds, and such calibration requires judgments about how to weigh false positives versus false negatives. These judgments are inescapably value judgments (Douglas, 2000).

6 There is, to be sure, a remaining problem. We have suggested that some norm change and structural change should be carried out in and by workplaces, and workplaces are not open to democratic control. This is a particular instance of a much more general problem: the extent to which workplaces exercise sweeping and unaccountable control over their employees (Anderson, 2017). We recognize this is a serious problem, but we take it neither to be specific to our proposals nor worsened by them.

7 When public resistance should lead to abandoning a policy and when politicians should attempt to implement it in the face of public resistance is a difficult issue. Support for capital punishment, at least as measured by surveys, remains high in many countries which have ended the practice; many ethicists would maintain that in this case politicians are right to resist public pressure. Nevertheless, there should be a presumption that the public settles policy. We suggest that proposals like those we outline might be implemented whether or not there is widespread public support for them, and consideration given to abandoning them if and when public support remains low.

## References

[phab008-B2] Anderson E. 2017. Private Government. Princeton, NJ: Princeton University Press.

[phab008-B3] Ballester J., Robine J.-M., Herrmann F. R., Rodó X. (2019). Effect of the Great Recession on Regional Mortality Trends in Europe. Nature Communications, 10, 10. 10.1038/s41467-019-08539-w PMC6368579 30737401

[phab008-B4] Bambery B., Douglas T., Selgelid M. J., Maslen H., Giubilini A., Pollard A. J., Savulescu J. (2018). Influenza Vaccination Strategies Should Target Children. Public Health Ethics, 11, 221–234. 30135702 10.1093/phe/phx021 PMC6093440

[phab008-B5] Beck L. F., Kresnow M., Bergen G. (2019). Belief about Seat Belt Use and Seat Belt Wearing Behavior among Front and Rear Seat Passengers in the United States. Journal of Safety Research, 68, 81–88. 30876523 10.1016/j.jsr.2018.12.007 PMC6422166

[phab008-B6] Betsch C., Böhm R., Chapman G. B. (2015). Using Behavioral Insights to Increase Vaccination Policy Effectiveness. Policy Insights from the Behavioral and Brain Sciences, 2, 61–73.

[phab008-B7] Bostrom N., Ord T. (2006). The Reversal Test: Eliminating Status Quo Bias in Applied Ethics. Ethics, 116, 656–679. 17039628 10.1086/505233

[phab008-B8] Bovens L. 2008. The Ethics of Nudge. In Preference Change: Approaches from Philosophy, Economics and Psychology, ed. Hansson M. J. and Grüne-Yanoff T., 207–20. Berlin: Springer.

[phab008-B59] Brewer, T. and Pringle, Y. (2015). Beyond Bazalgette: 150 years of sanitation. *The Lancet* 386 (9989), 128–129. 10.1016/S0140-6736(15)61231-4 26194385

[phab008-B9] Castilla J., Navascués A., Fernández-Alonso M., Reina G., Albéniz E., Pozo F., Álvarez N., Martínez-Baz I., Guevara M., García-Cenoz M., Irisarri F., Casado I., Ezpeleta C. and Primary Health Care Sentinel Network and Network for Influenza Surveillance in Hospitals of Navarra. (2016). Effects of Previous Episodes of Influenza and Vaccination in Preventing Laboratory-Confirmed Influenza in Navarre, Spain, 2013/14 Season. Eurosurveillance, 21, 30243. 10.2807/1560-7917.ES.2016.21.22.30243 27277013

[phab008-B10] CDC. 2019. Estimated Influenza Illnesses, Medical Visits, Hospitalizations, and Deaths in the United States—2017–2018 Influenza Season. Atlanta, GA: CDC.

[phab008-B11] CDC. 2020a. Coronavirus Disease 2019 (COVID-19). Atlanta, GA: Centers for Disease Control and Prevention.

[phab008-B12] CDC. 2020b. Estimated Influenza Illnesses, Medical Visits, Hospitalizations, and Deaths in the United States—2018–2019 Influenza Season. Atlanta, GA: CDC.

[phab008-B13] Cellan-Jones R., Kelion L., 2020. The great coronavirus-tracing apps mystery. *BBC News*, July 22.

[phab008-B14] Cho H., Ippolito D., Yu Y. W. 2020. Contact Tracing Mobile Apps for COVID-19: Privacy Considerations and Related Trade-offs.

[phab008-B15] Coady C. A. J. 1992. Testimony: A Philosophical Study. Oxford: Clarendon Press.

[phab008-B16] Department of Health and Social Care. 2020. Most comprehensive flu programme in UK history will be rolled out this winter. *GOV.UK*. July 24.

[phab008-B17] Dew K., Keefe V., Small K. (2005). ‘Choosing’ to Work When Sick: Workplace Presenteeism. Social Science & Medicine, 60, 2273–2282. 15748675 10.1016/j.socscimed.2004.10.022

[phab008-B18] Diekema D. S. (2014). Personal Belief Exemptions from School Vaccination Requirements. Annual Review of Public Health, 35, 275–292. 10.1146/annurev-publhealth-032013-182452 24328988

[phab008-B19] Dietz C., Zacher H., Scheel T., Otto K., Rigotti T. (2020). Leaders as Role Models: Effects of Leader Presenteeism on Employee Presenteeism and Sick Leave. Work & Stress, 34: 300–322.

[phab008-B61] Douglas, H. (2000). Inductive Risk and Values in Science. *Philosophy of Science*, **67**, 559–579.

[phab008-B20] Du L., Huang G. 2020. Japan May Have Beaten Coronavirus Without Lockdowns or Mass Testing. But How? *Time*, May 25.

[phab008-B22] Garrow V. 2016. *Presenteeism: A Review Of Current Thinking*. Brighton, UK: Institute for Employment Studies.

[phab008-B23] Giubilini A. 2019. The Ethics of Vaccination. In Brooks T. (ed.), Palgrave Studies in Ethics and Public Policy. London, UK: Palgrave Pivot. 30888744

[phab008-B24] Giubilini A., Caviola L., Maslen H., Douglas T., Nussberger A.-M., Faber N., Vanderslott S., Loving S., Harrison M., Savulescu J. (2019). Nudging Immunity: The Case for Vaccinating Children in School and Day Care by Default. HEC Forum, 31, 325–344. 31606869 10.1007/s10730-019-09383-7 PMC6841646

[phab008-B25] Giubilini A., Douglas T., Savulescu J. (2018). The Moral Obligation to Be Vaccinated: Utilitarianism, Contractualism, and Collective Easy Rescue. Medicine, Health Care and Philosophy, 21, 547–560. 10.1007/s11019-018-9829-y PMC6267229 29429063

[phab008-B26] Henry C., Zheng N.-Y., Huang M., Cabanov A., Rojas K. T., Kaur K., Andrews S. F., Palm A.-K. E., Chen Y.-Q., Li Y., Hoskova K., Utset H. A., Vieira M. C., Wrammert J., Ahmed R., Holden-Wiltse J., Topham D. J., Treanor J. J., Ertl H. C., Schmader K. E., Cobey S., Krammer F., Hensley S. E., Greenberg H., He X.-S., Wilson P. C. (2019). Influenza Virus Vaccination Elicits Poorly Adapted B Cell Responses in Elderly Individuals. Cell Host & Microbe, 25, 357–366.e6. 30795982 10.1016/j.chom.2019.01.002 PMC6452894

[phab008-B28] Jackson C., Vynnycky E., Hawker J., Olowokure B., Mangtani P. (2013). School Closures and Influenza: Systematic Review of Epidemiological Studies. BMJ Open, 3, e002149. 10.1136/bmjopen-2012-002149 PMC3586057 23447463

[phab008-B29] Jackson C., Vynnycky E., Mangtani P. (2016). The Relationship between School Holidays and Transmission of Influenza in England and Wales. American Journal of Epidemiology, 184: 644–651. 27744384 10.1093/aje/kww083 PMC5860259

[phab008-B30] Kinman G. (2019). Sickness Presenteeism at Work: Prevalence, Costs and Management. British Medical Bulletin, 129, 69–78. 30649219 10.1093/bmb/ldy043

[phab008-B31] Kinman G., Clements A. J., Hart J. (2019). When Are You Coming Back? Presenteeism in U.K. Prison Officers - Gail Kinman, Andrew James Clements, Jacqui Hart, 2019. The Prison Journal, 99, 363–383.

[phab008-B32] Kumar V. (2016). Nudges and Bumps. Georgetown Journal of Law and Public Policy, 14, 861–876.

[phab008-B33] Lackey J., Sosa E. (2006). The Epistemology of Testimony. Oxford, UK: Oxford University Press.

[phab008-B34] Levy N. (2019). Nudge, Nudge, Wink, Wink. Nudging is Giving Reasons. Ergo: An Open Access Journal of Philosophy, 6, 10.3998/ergo.12405314.0006.010. PMC6774767 31579296

[phab008-B35] Lohaus D., Habermann W. (2019). Presenteeism: A Review and Research Directions. Human Resource Management Review, 29, 43–58.

[phab008-B36] Maruthappu M., Watkins J., Noor A. M., Williams C., Ali R., Sullivan R., Zeltner T., Atun R. (2016). Economic Downturns, Universal Health Coverage, and Cancer Mortality in High-Income and Middle-Income Countries, 1990–2010: A Longitudinal Analysis. The Lancet, 388, 684–695. 10.1016/S0140-6736(16)00577-8 27236345

[phab008-B38] Peeples L. (2019). How the Next Recession Could save Lives. Nature, 565, 412–415. 30675054 10.1038/d41586-019-00210-0

[phab008-B40] Pluhar E. B. (2010). Meat and Morality: Alternatives to Factory Farming. Journal of Agricultural and Environmental Ethics, 23, 455–468.

[phab008-B41] Postma M. J. (2008). Public Health Economics of Vaccines in The Netherlands: Methodological Issues and Applications. Journal of Public Health, 16, 267–273.

[phab008-B42] Putri W. C. W. S., Muscatello D. J., Stockwell M. S., Newall A. T. (2018). Economic Burden of Seasonal Influenza in the United States. Vaccine, 36, 3960–3966. 29801998 10.1016/j.vaccine.2018.05.057

[phab008-B43] Reeves A., McKee M., Stuckler D. (2014). Economic Suicides in the Great Recession in Europe and North America. The British Journal of Psychiatry, 205, 246–247. 24925987 10.1192/bjp.bp.114.144766

[phab008-B44] Robichaud P., Wieland J. W. (2017). Responsibility—The Epistemic Condition. Oxford, UK: Oxford University Press.

[phab008-B60] Saghai, Y. (2013). Salvaging the concept of nudge. *Journal of Medical Ethics*, **39**, 487–493. 10.1136/medethics-2012-100727 23427215

[phab008-B45] Savulescu J. 2020. Pandemic Ethics: Good Reasons to Vaccinate: COVID19 Vaccine, Mandatory or Payment Model? Journal of Medical Ethics, 47, 78–85. 33154088 10.1136/medethics-2020-106821 PMC7848060

[phab008-B46] Savulescu J., Isdale W. (2015). Three Proposals to Increase Australia’s Organ Supply. Monash Bioethics Review, 33, 91–101. 26169530 10.1007/s40592-015-0030-2

[phab008-B47] Snitow S., Brennan L. (2011). Reducing Drunk Driving-Caused Road Deaths: Integrating Communication and Social Policy Enforcement in Australia. In Cheng H., Kotler P., Lee (eds N., Social Marketing for Public Health. Burlington, MA: Jones & Bartlett Learning.

[phab008-B48] Støvring K. (2014). The Conservative Critique of the Enlightenment: The Limits of Social Engineering. The European Legacy, 19, 335–346.

[phab008-B49] Thaler R. H., Sunstein C. R. 2008. Nudge: Improving Decisions about Health, Wealth and Happiness. New Haven, CT: Yale University Press.

[phab008-B50] The Economist. Face-off over face-masks: Europe’s latest north-south split. *The Economist*, 8 July 2020.

[phab008-B51] Vallier K. (2016). On the Inevitability of Nudging. Georgetown Journal of Law & Public Policy, 14.

[phab008-B52] Verity R., Okell L. C., Dorigatti I., Winskill P., Whittaker C., Imai N., Cuomo-Dannenburg G., Thompson H., Walker P. G. T., Fu H., Dighe A., Griffin J. T., Baguelin M., Bhatia S., Boonyasiri A., Cori A., Cucunubá Z., FitzJohn R., Gaythorpe K., Green W., Hamlet A., Hinsley W., Laydon D., Nedjati-Gilani G., Riley S., van Elsland S., Volz E., Wang H., Wang Y., Xi X., Donnelly C. A., Ghani A. C., Ferguson N. M. (2020). Estimates of the Severity of Coronavirus Disease 2019: A Model-Based Analysis. The Lancet Infectious Diseases, 20, 669–677. 32240634 10.1016/S1473-3099(20)30243-7 PMC7158570

[phab008-B53] Wada K., Oka-Ezoe K., Smith D. R. (2012). Wearing Face Masks in Public during the Influenza Season May Reflect Other Positive Hygiene Practices in Japan. BMC Public Health, 12, 1065. 23227885 10.1186/1471-2458-12-1065 PMC3536629

[phab008-B54] Wang Q., Yue N., Zheng M., Wang D., Duan C., Yu X., Zhang X., Bao C., Jin H. (2018). Influenza Vaccination Coverage of Population and the Factors Influencing Influenza Vaccination in Mainland China: A Meta-Analysis. Vaccine, 36, 7262–7269. 30340886 10.1016/j.vaccine.2018.10.045

[phab008-B55] WHO. (2018). Influenza (Seasonal). Geneva, Switzerland: WHO.

[phab008-B56] Wilkinson T. M. (2013). Thinking Harder about Nudges. Journal of Medical Ethics, 39, 486–486. 23861245 10.1136/medethics-2013-101687

[phab008-B57] Wilson R., Scronias D., Zaytseva A., Ferry M.-A., Chamboredon P., Dubé E., Verger P. (2019). Seasonal Influenza Self-Vaccination Behaviours and Attitudes among Nurses in Southeastern France. Human Vaccines & Immunotherapeutics, 15, 2423–2433. 30829102 10.1080/21645515.2019.1587274 PMC6816369

[phab008-B58] Yasaka T. M., Lehrich B. M., Sahyouni R. (2020). Peer-to-Peer Contact Tracing: Development of a Privacy-Preserving Smartphone App. JMIR Mhealth and Uhealth, 8, e18936. 32240973 10.2196/18936 PMC7144575

